# Effect of Inorganic and Organic Nitrogen Sources and Biofertilizer on Murcott Mandarin Fruit Quality

**DOI:** 10.3390/life12122120

**Published:** 2022-12-15

**Authors:** Ahmed M. Fikry, Khadija S. Radhi, Mohammed A. S. Abourehab, Talaat A. M. Abou Sayed-Ahmed, Mohamed M. Ibrahim, Farid S. Mohsen, Nour A. Abdou, Ahmad A. Omar, Ibrahim Eid Elesawi, Mohamed T. El-Saadony

**Affiliations:** 1 Horticulture Department, Faculty of Agriculture, Zagazig University, Zagazig 44519, Egypt; 2 Department of Food Science and Nutrition, College of Science, Taif University, Taif 21944, Saudi Arabia; 3 Department of Pharmaceutics, College of Pharmacy, Umm Al-Qura University, Makkah 21955, Saudi Arabia; 4 Deciduous Fruit Research Department, Horticulture Research Institute, ARC, Giza 12511, Egypt; 5 Biochemistry Department, Faculty of Agriculture, Zagazig University, Zagazig 44519, Egypt; 6 Citrus Research and Education Center, IFAS, University of Florida, Lake Alfred, FL 33850, USA; 7 College of Life Science and Technology, Huazhong Agricultural University, Wuhan 430070, China; 8 Department of Agricultural Microbiology, Faculty of Agriculture, Zagazig University, Zagazig 44511, Egypt

**Keywords:** Murcott mandarin, fertilization, nitrogen source, biofertilizers, fruit quality, vitamin C

## Abstract

Mandarin ‘Murcott’ (*Citrus reticulata* Blanco) trees aged five years that were grafted onto lemon ‘Volkamer’ (*Citrus volkameriana*) rootstock and grown in sandy soil under a drip irrigation system were used in this study during the growing seasons of 2018 and 2019. Ten different fertilization treatments combining inorganic, organic, and biofertilization in a completely randomized block were performed. The results revealed that fertilizing ‘Murcott’ mandarin trees with 75% of the recommended dose (RD) of nitrogen as inorganic nitrogen (33.5% N) in the form of NH_4_NO_3_ + 25% of RD as organic nitrogen in the form of chicken manure (3% N) per tree per year without or with a biofertilizer (Effective Microorganisms, EM1) at 150 mL/tree increased the weight, size, pulp, and peels of mandarin fruit, as well as the fruit juice volume, juice volume/fruit, and vitamin C, but reduced the total acidity in both seasons. However, fertilizing ‘Murcott’ mandarin trees with 100% of RD as inorganic nitrogen increased the pulp/fruit ratio, and fertilizing with 25% of RD as inorganic nitrogen + 75% of RD as organic nitrogen + biofertilizer EM1 increased the peel/fruit ratio, peel thickness, and fruit firmness. Fertilizing ‘Murcott’ mandarin trees with 100% organic nitrogen + biofertilizer EM1 increased total soluble solids (TSS) and total sugar contents while producing the lowest nitrate (NO_3_) percentage in ‘Murcott’ mandarin fruit compared with trees fertilized with inorganic nitrogen only. The fruit produced by ‘Murcott’ mandarin trees fertilized with 100% of RD as organic nitrogen with or without biofertilizer EM1 contained higher TSS, total carbohydrates, and sugars and lower nitrate percentages than those fertilized with inorganic nitrogen and biofertilizer EM1. This study contributes to reducing the use of inorganic fertilizers by adding a percentage of an organic fertilizer to obtain a healthy product that contains a lower percentage of NO_3_, which affects the health of the consumer, and is of high quality and suitable for export.

## 1. Introduction

The ‘Murcott’ mandarin (*Citrus reticulata* Blanco) is one of Egypt’s new major exportable fruit crops. It is reported that the USDA citrus breeding program in Florida produced the ‘Murcott’ mandarin through hybridization between a tangerine and a sweet orange in 1916. The fruit is medium-sized when the tree is carrying a moderate fruit load. The peel is yellowish-orange, and the flesh is deep orange at maturity. The rind is thin and smooth and peels moderately well. The ‘Murcott’ mandarin is considered seedy, with about 10–20 per fruit, and the commercial harvest season is from January to March [1]. It is estimated that the production of tangerines, mandarins, clementines, and satsumas in 2019 in Egypt was 1.1 million tons, with Egypt ranked fifth among the top ten producing countries [2].

For optimal vegetative growth, high fruit yield, and high-quality citrus fruit, nitrogen (N) fertilization is critical. Nitrogen is essential in nutrition because it is highly required by plants. It is important for plants because it is structurally involved in most catalytic biomolecules, such as proteins, nucleic acids, vitamins, hormones, and chlorophyll pigments [3]. Applying 600–1000 g N/year to mandarin trees improved fruit quality characteristics such as the juice content, total soluble solids (TSS), acidity, fruit size, and fruit dimensions [4].

Heavy inorganic fertilizers are used in Egypt, and these chemical fertilizers cause many problems for human health and microorganisms’ activity in the soil. Thus, organic fertilizers have been recommended to improve soil fertility, increase microorganisms’ activity in the soil, facilitate nutrient acquisition, and increase citrus tree productivity [5,6,7,8]. The application of organic manure as a source of organic nitrogen has many benefits, such as increasing soil fertility, organic matter content, water retention, nutrient availability, and soil cation exchange and reducing soil pH and salinity, as well as reducing nutrient losses and increasing the number of microbes that produce natural hormones and antibiotics that resist pathogenic microbes [6,7,8,9,10,11]. In this study, we chose chicken manure because several chicken farms are next to citrus trees; therefore, it is used as organic fertilizer. Chicken manure has a high mineral content, i.e., phosphorous, potassium, sulfur, and calcium. In addition, it is relatively rich in nitrogen content, with about 40–90% nitrogen, which exceeds the % N in animal manure by 3–5%, and produces the lowest percentage of nitrates in the crop while reducing the use of mineral fertilizers [12,13,14]. Moreover, it has a low nitrogen cost per pound [15]. Chicken manure is a cost-effective slow-release fertilizer because it incorporates plant nutrients in organic and inorganic forms. Nutrients found in inorganic forms can be readily available to plants. In contrast, organic nutrients become available as the manure decomposes, and the nutrients are available until the next season, compared to other low-release fertilizers [16].

Biofertilization is an effective method for improving citrus trees’ yield and fruit quality and has become a positive alternative to chemical fertilizers. They are safe for humans, animals, and the environment. Biofertilizers are recommended to reduce soil and underground pollution in our atmosphere and enhance organic food production for export [9]. Effective Microorganisms (EM1) are liquid biofertilizers containing many beneficial microorganisms, such as photosynthetic bacteria, fungi, actinomycetes, lactic acid bacteria, and yeast. They are mainly used to restore healthy soil and water [17]. EM1 treatments improved soil chemical and physical conditions, enhancing the yield and fruit quality of citrus trees [18]. The positive effects of bio- and organic fertilizers on ‘Balady’ mandarin trees could be mainly due to their ability to adequately supply trees with their requirements for various nutrients for a relatively long time, as well as reduce nitrite pollution and produce organic fruit with higher quality [19]. No studies have investigated the effect of mixing three fertilizers on the quality of Murcott trees. Therefore, this study aimed to investigate the effects of replacing inorganic nitrogen fertilization with organic nitrogen and biofertilizers. To determine the best mixture, three fertilizers were mixed, namely, inorganic (25–100%), organic (25–100%), and biological fertilizers (150 mL), to determine the best conditions while monitoring the physiochemical quality attributes of ‘Murcott’ mandarin fruit.

## 2. Materials and Methods

### 2.1. Plant and Soil Conditions

The experiment was conducted on 5-year-old ‘Murcott’ mandarin trees (*Citrus reticulata*, Blanco) grafted on the rootstock of ‘Volkamer’ lemon (*Citrus volkameriana*) over two consecutive growing seasons in 2018 and 2019 on a private farm (Latitude: N 30°56′11.62″; Longitude: E 31°50′35.635″). The experimental trees had the same growth vigor, size, and health and were planted under a drip irrigation system in sandy soil at 2.5 × 5 m^2^. They were subjected to standard agro-technical practices of irrigation and pruning for commercial citrus orchards and pest control. In addition, all trees received 475 Kg/ha of calcium superphosphate (15.5% P_2_O_5_) and 475 Kg/ha of potassium sulfate (48.5% K_2_O).

The tested soil and chicken manure samples were analyzed for their chemical and physical properties (sandy-texture sand, 92.51%; silt, 6.22%; clay, 1.27%). The physiochemical properties of soil and chicken manure are shown in Table 1. The physiochemical analysis of chicken manure was conducted as per Huang et al. [20], and the soil analysis was performed following de Sousa Lima et al. [21].

### 2.2. Fertilization Treatments

Fifty ‘Murcott’ mandarin trees were treated with different fertilizers in ten treatments with five replicates for each treatment (Table 2). Data were collected from three replicates in the middle of each treatment regarding doses of inorganic nitrogen fertilization in the form of ammonium nitrate (33.5% nitrogen), organic nitrogen in the form of chicken manure (3% N), and a biofertilizer (Effective Microorganisms (EM1)). EM1 is a liquid biofertilizer containing many beneficial microbes for plants [17]. In both seasons of the study, the treatments described in Table 2 were applied to the same trees each season.

Throughout the first and second seasons, all selected trees were fertilized weekly with the inorganic nitrogen source from February 15th until September 15th. The recommended doses (RDs) of nitrogen (100%) equaled 1.492 and 1.791 kg of ammonium nitrate (33.5% N) as inorganic nitrogen and 16.66 and 20 kg of chicken manure (3%) as organic nitrogen in the 1st and 2nd seasons, respectively [22,23,24]. The RDs of nitrogen for the first and second seasons were about 0.5 and 0.6 kg nitrogen/tree/year. The organic fertilizer, chicken waste at a concentration of 30,000 ppm, was spread once under drippers and promptly covered with moist soil. The same quantity of chicken manure was utilized in both seasons to avoid compositional changes. The physical and chemical properties of chicken manure are listed in Table 1.

The EM1 biofertilizer was added at 0.15 L/tree. Another treatment prepared by homogenizing EM1 (0.15 L) in 5 L of water was added at 150 mL per tree. The biofertilizer EM1 amount was mixed with 5 L of water and placed on the chicken manure in a trench of 10 cm depth. EM1 was obtained from the Egyptian Ministry of Agriculture, Dokki, Giza, Egypt. The trees were irrigated with 12 and 16 M^3^ water/tree/year during the two seasons.

The recommended doses (RDs) of nitrogen (100%) equaled 1.492 and 1.791 kg/tree ammonium nitrate (33.5%N) as inorganic nitrogen and 16.66 and 20 kg/tree chicken manure (3%) as organic nitrogen in the 1st and 2nd seasons, respectively. The RDs of nitrogen were about 500 and 600 g/tree nitrogen/tree/year in the 1st and 2nd seasons, respectively.

### 2.3. Data Collection

After fruit harvest in the first week of February, to determine the following fruit characteristics, 15 fruits per tree were collected randomly from three trees, eliminating the first and last trees of each treatment (a total of 45 fruits per treatment):

*Fruit physical parameters:*

Fruit weight (g) and size (cm^3^), pulp and peel weights (g), fruit firmness (g/cm^2^), peel thickness (mm), pulp, and the peel/fruit ratio were measured. The average volume of juice/fruit (cm^3^) was estimated by juicing ten fruits from each treatment.

*Juice chemical characteristics:*

After extracting the pulp and pressing it using an Electric Extractor to extract the juice, the following chemical characteristics were estimated: Total soluble solids (TSS%) were measured using a hand refractometer in fruit juice. Then, the ratio of TSS/acid was determined. The titratable acidity (TA) percentage, vitamin C content, total carbohydrates, and sugar percentages were estimated according to AOAC [25]. The nitrate percentage was determined in dry fruit pulp tissue based on standards of the Association of Analytical Communities (AOAC) and the International Organization for Standardization (ISO) [25,26].

### 2.4. Statistical Analysis

#### Statistical Examination

In this study, a completely randomized block design (CRBD) was used, with 10 treatments and 5 replications of each treatment. A one-way ANOVA test was used to analyze the data using Co-Stat version 6.45 based on Snedecor and Cochran [27]. Duncan’s multiple range test with a significance level of 0.05 was used to compare the means [28].

## 3. Results

### 3.1. Physical Fruit Characteristics

#### 3.1.1. Fruit Weight and Size

The results indicated that the weight and size of ‘Murcott’ mandarin fruit was significantly impacted by the added fertilizers tested during the seasons (Table 3). The highest weight and size of the fruit were recorded for 75% inorganic nitrogen + 25% organic nitrogen, followed by fertilization with 50% inorganic nitrogen + 50% organic nitrogen + biofertilizer EM1 (Table 3). ‘Murcott’ mandarin trees fertilized with 25% inorganic nitrogen + 75% organic nitrogen + biofertilizer EM1 produced the lowest fruit weight and size in both seasons compared with the other treatments.

Fruit weight (FW), fruit size (FS), pulp weight (PW), and peel weight (PEW) were measured for recommended doses (RDs) of nitrogen (100%) equal to 1.492 and 1.791 kg of ammonium nitrate (33.5%N) as inorganic nitrogen and 16.66 and 20 kg of chicken manure (3%) as organic nitrogen. The RDs of nitrogen were about 0.5 and 0.6 Kg N/tree/year in the first and second seasons, respectively.

#### 3.1.2. Pulp and Peel Weights

The highest fruit pulp and peel weights were noticed in trees fertilized with 75% of RD as inorganic nitrogen + 25% of RD organic nitrogen, followed by those fertilized with 50% inorganic nitrogen + 50% organic nitrogen + biofertilizer EM1 (Table 3). The lowest weight of fruit pulp was produced by trees fertilized with 25% of RD as inorganic nitrogen + 75% of RD as organic nitrogen + biofertilizer EM1 and those fertilized with 100% of RD as organic nitrogen, while the lowest weight of peel was produced by trees fertilized with 50% inorganic nitrogen + 50% organic nitrogen (Table 3).

#### 3.1.3. Juice Volume/Fruit

‘Murcott’ mandarin trees fertilized with 75% inorganic nitrogen + 25% organic nitrogen achieved the highest amount of juice/fruit, whereas the lowest juice volume was found in trees fertilized with 100% organic nitrogen + biofertilizer EM1 in both seasons (Table 3). From the previous results, it could be concluded that fertilizing ‘Murcott’ mandarin trees with 75% of RD as inorganic nitrogen (33.5% N) in the form of NH_4_NO_3_ + 25% of RD as organic nitrogen in the form of chicken manure (3% N) without or with the biofertilizer (Effective Microorganisms, EM1) at 150 mL/tree increased the weight, size, pulp, and peels of mandarin fruit as well as the fruit juice volume. Compared with the other fertilizer treatments, the fertilizer treatment using organic nitrogen and the biofertilizer showed good values for the juice volume/fruit in both seasons (Table 3).

#### 3.1.4. Pulp and Peel/Fruit Ratio

**Figure 1:**
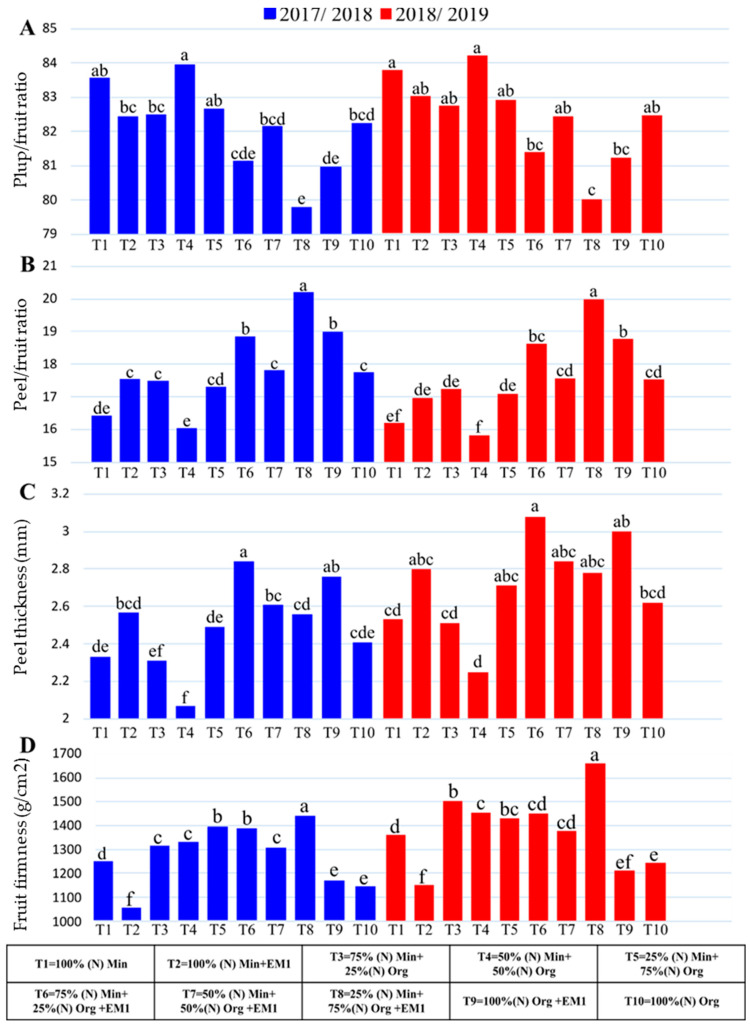
Effects of inorganic and organic nitrogen with EM1 extract on (**A**) pulp/fruit ratio, (**B**) peel/fruit ratio, (**C**) peel thickness (mm), and (**D**) fruit firmness (g/cm^2^) of ‘Murcott’ mandarin fruit during the 2018 and 2019 seasons. Different lowercase letters (a–f) indicate significant differences (*p* ≤ 0.05).

In both seasons, the fertilizer treatments significantly affected the pulp and peel/fruit ratio (Figure 1A and Figure 1B, respectively). ‘Murcott’ mandarin trees fertilized with 50% of RD as inorganic nitrogen + 50% of RD as organic nitrogen and those fertilized with only 100% inorganic nitrogen had the highest pulp/fruit ratio in both seasons, while the lowest pulp/fruit ratio resulted from trees fertilized with 25% inorganic nitrogen + 75% organic nitrogen + biofertilizer EM1 in both seasons (Figure 1A). The peel/fruit ratio of ‘Murcott’ mandarin fruit fertilized with 25% inorganic nitrogen + 75% organic nitrogen + biofertilizer EM1 produced the highest values, while the lowest values of the peel/fruit ratio resulted from trees fertilized with 50% inorganic nitrogen + 50% organic nitrogen and 100% inorganic nitrogen (Figure 1B).

The recommended doses (RDs) of nitrogen (100%) equaled 1.492 and 1.791 kg of ammonium nitrate (33.5%N) as inorganic nitrogen and 16.66 and 20 kg of chicken manure (3%) as organic nitrogen in the first and second seasons, respectively. The RDs of nitrogen were about 0.5 and 0.6 Kg N/tree/year in the first and second seasons, respectively.

#### 3.1.5. Peel thickness

‘Murcott’ mandarin fruit produced by trees fertilized with 75% of RD as inorganic nitrogen + 25% of RD as organic nitrogen + biofertilizer EM1 had the maximum peel thickness in both seasons, with no significant differences obtained with 100% organic nitrogen + biofertilizer EM1 and 50% inorganic nitrogen + 50% organic nitrogen + biofertilizer EM1 (Figure 1C). These treatments resulted in good growth and nutritional status, especially for calcium content in ‘Murcott’ mandarin leaves, consequently increasing the peel thickness of the fruit, thus enhancing the pulp, peel, and fruit firmness. These results may also be attributed to the fact that organic and biofertilizers help facilitate the availability and uptake of most nutrients in the trees. The obtained results agree with Shaimaa and Massoud [29], who found that ‘Washington’ navel orange trees fertilized with 75% of RD as inorganic nitrogen + 25% of RD as organic nitrogen + biofertilizer EM1 produced fruit with the thickest peel and a higher peel weight, while trees fertilized with 50% inorganic nitrogen + 50% organic nitrogen induced the thinnest fruit peel. In contrast, Abedel-Sattar et al. [30] indicated that ‘Washington’ navel orange trees fertilized with various nitrogen sources (inorganic and organic) and biofertilizers produced the highest peel thickness.

#### 3.1.6. Fruit Firmness

‘Murcott’ mandarin trees fertilized with 25% inorganic nitrogen + 75% organic nitrogen + biofertilizer EM1 had the highest fruit firmness, while trees fertilized with 100% inorganic nitrogen had the lowest fruit firmness in both seasons (Figure 1D). From the initial results, it could be concluded that fertilizing ‘Murcott’ mandarin trees with 100% of RD as inorganic nitrogen increased the pulp/fruit ratio, and fertilizing with 25% of RD as inorganic nitrogen + 75% of RD as organic nitrogen + biofertilizer EM1 increased the peel/fruit ratio, peel thickness, and fruit firmness (Figure 1D).

Combining chicken manure with inorganic fertilizer achieves the same trend; in our study, the mixture of chicken manure and inorganic fertilizer in a 75:25 ratio achieved the optimal requirements for mandarin fruit. These results agree with Bhatnagar et al. [31] and Hijbeek et al. [32].

### 3.2. Physiochemical Properties of ‘Murcott’ Mandarin Fruit

#### 3.2.1. Total Soluble Solids Percentage (TSS%)

‘Murcott’ mandarin trees fertilized with 100% of RD as organic nitrogen, 100% of RD as inorganic nitrogen without EM1, and 100% of RD as organic nitrogen + biofertilizer EM1 produced the greatest percentage of TSS in both seasons, with no significant differences obtained with fruit produced by trees fertilized with 25% inorganic nitrogen + 75% organic nitrogen + biofertilizer EM1 in the first season. The lowest percentage of TSS was in fruit produced by trees fertilized with 50% inorganic nitrogen + 50% organic nitrogen + biofertilizer EM1 in both seasons (Table 4).

Total acidity (TA) was measured for recommended doses (RDs) of nitrogen (100%) equal to 1.492 and 1.791 kg of ammonium nitrate (33.5%N) as inorganic nitrogen and 16.66 and 20 kg of chicken manure (3%) as organic nitrogen in the first and second seasons, respectively. The RDs of nitrogen were about 500 and 600 g nitrogen/tree/year in the first and second seasons, respectively.

#### 3.2.2. Total Acidity Percentage

The fertilization treatments in both seasons significantly affected the total acidity percentage in the fruit juice. Nevertheless, the highest total acidity percentage was recorded for fruit produced by trees fertilized with 100% inorganic nitrogen only, 25% inorganic nitrogen + 75% organic nitrogen, and 25% inorganic nitrogen + 75% organic nitrogen + biofertilizer EM1 in both seasons (Table 4). The lowest total acidity percentages were obtained for fruit produced by trees fertilized with 100% organic nitrogen in the first season and for fruit produced by trees fertilized with 100% organic nitrogen + biofertilizer EM1 in the second season, while fruit produced by trees fertilized with 75% inorganic nitrogen + 25% organic nitrogen + biofertilizer EM1 obtained lower total acidity in both seasons (Table 4).

#### 3.2.3. TSS/Acid Ratio

The effect of the fertilization treatments on the TSS/acid ratio was approximately the opposite of their impact on the total acidity percentage in both seasons. ‘Murcott’ mandarin fruit produced by trees fertilized with 100% of RD as organic nitrogen in the first season and fruit produced by trees with 100% of RD as organic nitrogen + biofertilizer EM1 in the second season showed the maximum TSS/acid ratio (Table 4). Fruit produced by trees fertilized with 25% inorganic nitrogen + 75% organic nitrogen achieved low TSS/acid ratios in the first season, while fruit produced by trees fertilized with 75% inorganic nitrogen + 25% organic nitrogen showed the lowest TSS/acid ratios in the second season (Table 4).

#### 3.2.4. Content of Vitamin C (Ascorbic Acid)

‘Murcott’ mandarin fruit produced by trees fertilized with 100% inorganic nitrogen + biofertilizer EM1 in the first season and that produced by trees fertilized with 75% inorganic nitrogen + 25% organic nitrogen + biofertilizer EM1 in the second season had a higher content of vitamin C (Table 4). Fruit produced by trees fertilized with 100% organic nitrogen and 100% organic nitrogen + biofertilizer EM1 contained the lowest vitamin C content in the first season but without significant differences when compared with fruit produced by trees fertilized with 25% inorganic nitrogen + 75% organic nitrogen in the second season (Table 4).

#### 3.2.5. Nitrate Percentage in Fruit

The obtained results in Table 4 show that the percentage of nitrate (NO_3_) content in the ‘Murcott’ mandarin fruit tissue was severely affected by the nitrogen fertilization treatments in both seasons. The fruit on trees fertilized with 100% inorganic nitrogen and that on trees with 75% inorganic nitrogen + 25% organic nitrogen + biofertilizer EM1 had the maximum nitrate concentration in both seasons (Table 4). In contrast, the fruit produced by trees fertilized with 100% organic nitrogen + biofertilizer EM1 contained the minimum NO_3_ concentrations in the fruit tissues in both seasons (Table 4).

#### 3.2.6. Total Sugar Content (Reducing and Non-Reducing Sugars)

‘Murcott’ mandarin fruit produced by trees fertilized with 100% organic nitrogen + biofertilizer EM1 contained the highest percentages of total carbohydrates, sugars, and reducing sugars in both seasons, whereas fruit produced by trees fertilized with 100% inorganic nitrogen without or with biofertilizer EM1 had the lowest rates in both seasons (Table 5). Additionally, organic and inorganic nitrogen treatments with biofertilizer EM1 exhibited higher percentages than those obtained with only 100% inorganic nitrogen for all parameters (Table 5).

‘Murcott’ mandarin fruit produced by trees fertilized with 50% inorganic nitrogen + 50% organic nitrogen + biofertilizer had the highest non-reducing sugar percentage in the first season, and fruit produced by trees that received 50% of RD as inorganic nitrogen + 50% of RD as organic nitrogen had the highest in the second season. In contrast, fruit produced by trees fertilized with 25% inorganic nitrogen + 75% organic nitrogen in the 2018 season and fruit produced by trees fertilized with 25% inorganic nitrogen + 75% organic nitrogen + biofertilizer EM1 in the second season had the lowest non-reducing sugar percentage (Table 5).

The recommended doses (RDs) of nitrogen (100%) equaled 1.492 and 1.791 kg of ammonium nitrate (33.5%N) as inorganic nitrogen and 16.66 and 20 kg of chicken manure (3%) as organic nitrogen in the first and second seasons, respectively. The RDs of nitrogen were about 0.5 and 0.6 Kg N/tree/year in the 2018 and 2019 seasons.

## 4. Discussion

Nitrogen (N) plays an essential role in tree nutrition for the consistent bearing and quality of citrus fruits, like other fruit crops [33,34]. Nitrogen has the most noticeable effect on overall citrus tree growth, yield, and fruit quality [35]. Nitrogen is a structural component of many organic substances, including proteins, vitamins, hormones, nucleic acids, chlorophyll, and other active components [3,36]. Nitrogen enhanced the fruit size, peel thickness, juice content, and tree yield in some citrus species [37,38]. Nitrogen is added to trees through various sources, including inorganic, organic, and biofertilizers.

In general, ‘Murcott’ mandarin trees fertilized with inorganic nitrogen, organic nitrogen, and biofertilizer EM1 produced the highest fruit weight and size compared to trees fertilized with inorganic and organic nitrogen without using biofertilizer EM1 in both seasons. Inorganic and organic nitrogen combined with biofertilizer EM1 had a beneficial effect on yield, fruit weight, and fruit size. Additionally, different nitrogen sources improve most fruit chemical quality characteristics, such as TSS, the acid ratio, vitamin C, nitrate content, and carbohydrates. This could be attributed to their positive effects on improving soil fertility; the activity of microflora; the availability of most nutrients; the uptake of water; the secretion of hormones such as Indole-3-acetic acid (IAA), gibberellic acid (GA_3_), and cytokinin; vitamin B; and the resistance of the trees to different diseases [39]. In the current study, there was an increase in fruit number/tree in the second season compared with the first season because of the availability of nutrients. These results agree with El-Shazly et al. [40] and Hadole et al. [41], who reported that using different nitrogen fertilizers increased fruit weight and height.

Inorganic nitrogen fertilizers have more advantages, such as increased yield, canopy volume, leaf N concentration, and the TSS content of the fruit [42]. However, inorganic N fertilizers have some disadvantages to human health, such as nitrate and nitrite retention in citrus fruits [43]. Consequently, natural organic materials have been used to significantly improve soil fertility and the productivity of fruit trees [44,45,46,47]. Thus, a primary focus is on decreasing excessive inorganic nitrogen fertilization, especially in sandy soil that is naturally poor in either nutrient elements or organic matter, by using alternative organic N fertilizers as well as biofertilizers, which have been shown to be more significant nitrogen sources for crop efficiencies and, in particular, fruit crops when inoculated with organic matter [48]. Organic nitrogen sources typically have low nitrogen content. Still, they have many advantages, which may improve soil fertility, organic matter content, soil texture, and soil-water-holding capacity. They thus can increase nutrient availability and improve pH in soils with inherently low water and nutrient retention capacities [35].

Biofertilization focuses on modifying rhizobacteria by inoculating the seeds or soil with certain organisms to induce beneficial effects on a compatible host [49]. Biofertilizers are physical preparations containing live or suppressed cells of efficient strains of nitrogen-fixing, phosphate-solubilizing, or cellulolytic microorganisms that accelerate specific microbial processes to augment the extent of the availability of nutrients in a form that plants can quickly assimilate [50,51]. Several activities other than nitrogen fixation may be responsible for these favorable benefits, such as the generation of growth regulators, protection from root infections, and changes in nutrient intake by the plant [52]. Using biofertilizers in combination with organic fertilizers enhances yield and helps in overcoming drought, salt, and some pathogen stresses, reducing the applied fertilizers, and increasing the availability of most macro- and microelements. Inoculation with biofertilizers reduces the regular inorganic nitrogen fertilizer content as well as stimulates plant production [53].

The good effect of various nitrogen fertilizer sources (inorganic, organic, and biofertilizers (EM1)) on physical fruit attributes may be attributed to their beneficial influence on the growth and nutritional condition of ‘Murcott’ mandarin trees. This positive effect significantly increased fruit weight, size, the number of fruits/tree, and yield when adding biofertilizer (EM1) to the organic nitrogen source (chicken manure). Combining a biofertilizer with organic nitrogen improved these fertilizers’ efficiencies in improving physical and chemical fruit characteristics (Figure 1 and Table 4). Its positive effects included enhancing soil fertility and biological activity, facilitating nutrient uptake, improving cell division, and producing excellent juice content [54].

The highly increased weight of ‘Murcott’ mandarin fruit pulp affected by different nitrogen fertilizer treatments was attributed to their positive effects on enhancing cell division, increasing the juice content, and increasing pulp and fruit weights. Shaimaa and Massoud [29] reported that ‘Washington’ navel orange trees fertilized with 75% of RD as inorganic nitrogen + 25% of RD as organic nitrogen and 75% inorganic nitrogen + 25% organic nitrogen + biofertilizer EM1 produced fruit with the highest pulp weight compared to the other treatments. These findings agree with those of Hazarika and Aheibam [55]. They reported that lemon (*Citrus limon* Burm.) trees fertilized with 75% of RD as inorganic nitrogen + 25% of RD as organic nitrogen produced the highest amount of juice per fruit.

These nitrogen sources resulted in good growth and nutritional status and exceptionally high calcium content in ‘Murcott’ mandarin leaves, consequently increasing the peel thickness of ‘Murcott’ fruit and enhancing pulp, peel, and firmness. Moreover, these results may be attributed to the fact that organic and biofertilizers help to facilitate the availability and uptake of most nutrients, nutrient transport, the photosynthesis process, the fixation of nitrogen, antibiotic biosynthesis, water-use effectiveness, vitamin B, the solubility of most nutrients, soil workability, and resistance to drought [56,57,58].

The positive effects of organic manure and biofertilizer (EM1) are due to the many species of beneficial microbes they contain, such as lactic acid bacteria, photosynthetic bacteria, ray fungi actinomycetes, and yeast, boosting soil fertility, secreting natural hormones, and supplying plants with the minerals, amino acids, organic acids, and antioxidants they require [59,60,61]. Faissal et al. [62] reported the beneficial effects of using different nitrogen sources (inorganic, organic, and biofertilizers) on ‘Balady’ mandarin fruit characteristics, especially when combined with organic and biofertilizers.

Biofertilizers and organic fertilizers reduce the presence of salts in fruit juice, which leads to an increase in the percentage of TSS and an increase in the proportion of sugars in the fruit. Fruit quality in respect of TSS, total sugars, and ascorbic acid was significantly improved. This might be due to the improved vegetative development of the treated trees, which resulted in greater quantities of photosynthates (starch, carbohydrates, etc.) and their translocation to the fruit, hence boosting the values of different quality criteria in the fruits [63].

The tremendous effect of these nitrogen fertilizer sources on the quality of ‘Murcott’ mandarin fruit was attributed to their positive effects on enhancing cell division and building macromolecules, mainly carbohydrates. These findings align with those of Hadole et al. [41] and Garhwal et al. [64]. From the results obtained in the current study, it could be concluded that fertilizing with 100% of RD as organic nitrogen + biofertilizer EM1 increased TSS%, total carbohydrates, sugars, and reducing and non-reducing sugars and produced the lowest NO_3_ in ‘Murcott’ mandarin fruit.

The significant effects of decomposition and the transformation of inorganic nitrogen to the entire state are surely reflected in the reduced nitrate in the pulp of ‘Murcott’ mandarin fruit. The overuse of inorganic nitrogen fertilizer has been associated with increased nitrate-nitrogen levels (NO_3_–N) in groundwater and surface water, which influences the nitrate content of plants [65]. Therefore, high nitrate accumulation in plants harms human health [40]. For these reasons, developing a better system for recommending fertilizer rates is a primary aim of agricultural research because inorganic nitrogen fertilizers easily form nitrate, whereas organic fertilizers slowly form nitrate. These findings are in harmony with those reported by Kandel and Chhetri [4]. They reported that reducing the inorganic NPK source and increasing the organic or biofertilizer sources for mandarin trees improved the fruit quality and reduced the nitrite content in the juice.

The significant effects of using organic and biofertilizers for N led to reduced nitrate in the pulp of ‘Murcott’ mandarin fruit. Furthermore, the beneficial effect of these organic and biofertilizers on the reduced nitrate percentage might be attributed to their ability to decrease soil pH; additionally, the biofertilizer contains several helpful microorganisms, including photosynthetic bacteria, lactic acid bacteria, actinomycetes, and yeast, which increase soil fertility and organic matter, thereby increasing nutrient bioavailability and decreasing the retention of nitrate pollution in ‘Murcott’ mandarins [59,60,61]. These effects also enhanced the availability of various nutrients and reduced nitrate pollution retention in ‘Murcott’ mandarin fruit. Furthermore, organic and biofertilizers have beneficial effects by reducing nitrate and nitrite. Kandel and Chhetri [4] reported that an improvement in ‘Mandarin’ fruit quality with a reduction in juice nitrite content was associated with a decrease in the mineral NPK source and, at the same time, an increase in the organic or biofertilization sources.

Therefore, our findings are in accord with the importance of organic wastes. Chicken manure may be valorized by anaerobic digestion, characterized by odor reduction, greenhouse gas mitigation, the generation of gaseous biofuel, the prevention of the eutrophication of water bodies, and a great enhancement in global renewable energy production to assist nations in lowering their carbon emissions and other gases [31,32]. However, there are some concerns about chicken manure containing antibiotic-resistant microbes, such as *Escherichia coli*, *Listeria*, *Salmonella*, *Staphylococcus*, *Campylobacter*, *Clostridium*, *Actinobacillus*, *Bordetella*, *Corynebacterium*, *Globicatella*, *Mycobacterium*, and *Streptococcus*, as well as high contents of heavy metals, but this can be overcome as follows.

Concerning the heavy-metal content, Ravindran et al. [66] found that Cr, Cu, Ni, Pb, and Zn were present in chicken manure, and they were below the maximum permissible limits set by the US Environmental Protection Agency. However, regarding the microbial content, composting is often used as a disease management method to recycle animal feces into the soil to increase fertility [67]. Heating is advised to decrease or eradicate possible bacterial pathogens in animal manure after or before composting.

Meanwhile, various physical, chemical, and biological disinfection procedures for animal waste processing have been developed. The demand for bio-composting has recently increased to treat animal waste in an eco-friendly manner, and the trend toward organic farming has also increased [68,69,70,71,72]. Bio-compost is produced through the bio-oxidation of animal waste into stable products free of pathogenic microbes, in contrast to fresh compost, and is easy to apply to the soil [73]. In addition, composting’s initial capital, operational, and maintenance expenses are less than those of other treatment methods [74]. Thus, composting is an efficient method for adding value to poultry waste for agricultural purposes.

Build-up is a common way to keep bulk animal waste, or it can be utilized as fodder until it can be composted or put in fields. During the growth season of broilers, chicken litter often accumulates in the chicken house before its land application. Because the temperature required to reduce or eliminate bacterial populations is not attained in deeper layers, the middle and bottom areas of the built-up broiler litter bed provided less favorable conditions for anaerobic bacteria and coliforms than the top region [75].

Waste compost is a regulated method of combining organic wastes with additional materials in optimal proportions to maximize microbial development [76]. Composting is the biological digestion of organic wastes by a consortium of microbes in an aerobic atmosphere. It is a fast bio-degradation process that converts organic waste into stable and useful organic chemicals in approximately 4–6 weeks of microbial activity. Composting removes plant and human pathogens, reduces the waste volume, and eliminates weed seeds. However, its limitations include losing nitrogen and other minerals during the composting process, incurring installation and staffing charges, having a strong odor, and needing enough storage and operating space [73].

The expense of transporting chicken litter is a substantial barrier to a more practical application of this poultry byproduct. The more significant bulk density of chicken litter from composting can lower shipping costs. To reduce ammonia volatilization during composting, producers add certain additives to chicken waste, i.e., aluminum sulfate, straw, woodchip, paper waste, peat, and zeolite [77]. Additionally, although composting produces a stable waste product, some heavy metals, such as Cd, Zn, and Cu, may be present in chicken manure byproducts but still in the proper concentrations [78].

Composting goes through four main stages, depending on microbial population (mesophilic or thermophilic), temperature, cooling, and maturation [78]. Aerobic microbial composting raises the compost temperature to the thermophilic zone, between 45 and 75 °C. A well-managed compost operation should attain temperatures between 55 and 65 °C [79], which is suitable for eliminating mesophilic microbes, including *Salmonella* spp. and *Escherichia coli* O157:H7 [80].

No government authorities currently regulate the composting of animal feces in the United States. In 7 CFR 205.2 of the National Organic Program, supervised by the USDA, composting criteria are provided to improve soil fecundity, which is required for USDA Certified Organic recognition [80]. The proposed requirements are related to two scientifically sound, regulated composting processes: (1) composting that is stable and maintains aerobic conditions at 55 °C and controlled pH for three days, followed by appropriate treatment, and (2) dynamic composting. However, the USDA guidelines recommend slightly different temperature and time criteria for composting dead poultry [81] and state that a temperature > 50 °C for five days is sufficient to eliminate pathogens in the compost.

As a result of the continuous increase in poultry, their waste is valorized by producing organic fertilizers, where soluble nutrients are converted into stable organic materials. The composting process can sometimes eradicate common bacteria that cause foodborne illnesses. Multiple studies have shown that composting poultry litter successfully reduces foodborne germs. In 64 composted chicken litter samples, no *E. coli* O157:H7 or *Salmonella* spp. were found.

Pathogenic bacteria such as *Campylobacter jejuni*, *Salmonella* spp., and *Listeria monocytogenes* in chicken compost were eliminated when the temperature approached 55 °C [82]. After one week, chicken manure infested with *Campylobacter perfringens* and *Salmonella* was collected to measure the number of surviving bacteria, and the authors found that these pathogens were eradicated from chicken manure samples [83]; however, they were still recoverable from the undecomposed samples. In addition, Guan et al. [84] discovered that composting chicken dung at sufficiently high temperatures might diminish or destroy heat-sensitive genetically engineered *Pseudomonas chlororaphis* and its transgenes. Silva et al. [85] found that the treated manure was free of fecal coliform groups and *Salmonella* spp. The compost pile was not found to include a thermophilic phase (temperature > 40 °C). According to the literature on composting chicken waste, composting is a practical and ecologically friendly way to decrease or remove germs that cause foodborne illnesses. In addition, it should be stressed that adequate composting management is required to guarantee that the process reaches the desired time–temperature combination for pathogen eradication.

## 5. Conclusions

‘Murcott’ mandarin fruit produced by trees fertilized with 75% of RD as inorganic nitrogen + 25% of RD as organic nitrogen without or with biofertilizer EM1 improved fruit weight, juice volume, juice volume/fruit, and vitamin C but reduced total acidity in both seasons. The fruit produced by trees fertilized with 100% organic nitrogen with or without biofertilizer EM1 contained higher TSS, total carbohydrates, sugars, and lower nitrate percentages than those produced by trees fertilized with inorganic and biofertilizers. From the current study, it could be concluded that supplying ‘Murcott’ mandarin trees with 75% of RD as inorganic nitrogen + 25% of RD as organic nitrogen with or without biofertilizer EM1 could improve yield quantitatively and qualitatively and prevent environmental pollution. This study recommends reducing the use of inorganic fertilizers while still producing a high-quality product that is appropriate for export and has a reduced percentage of NO_3_, which is harmful to consumer health, by including a percentage of organic fertilizers for citrus fruit, which is considered a strategic crop for export.

## Figures and Tables

**Table 1:** Physiochemical analysis of soil and chicken manure.

Soil Analysis		Chicken Manure Analysis	
Characteristic	Values	Characteristic	Values
Physiochemical Properties			
Sand	92.51 ± 0.2	pH	6.70
Silt	6.22 ± 0.1	Organic C (%)	22.11
Clay	1.27 ± 0.3	C/N ratio	7.45
Texture class	Sand	N *	3.10
CEC, cmolckg^−1^	3.55 ± 0.1	K *	16,700
EC_e_, dSm^−1^	0.79 ± 0.06	P *	13,300
pH (1:2.5)	8.40 ± 0.2	Ca *	8500
OM, gkg^−1^	1.32 ± 0.1	Cu *	18.1 × 10^4^
CaCO_3_, gkg^−1^	19.10 ± 0.3	Mg *	5900
Soluble ions, mmol L^−1^		As *	13,100
Ca++	3.09 ± 0.1	Zn *	9 × 10^5^
Na^+^	3.72 ± 0.2		
Mg++	0.96 ± 0.01		
K^+^	0.95 ± 0.03		
Cl^−^	2.11 ± 0.1		
HCO_3_^−^	1.93 ± 0.9		
SO_4_^2−^	4.52 ± 0.5		

* Indicates values with ppm; N = nitrogen; P = phosphate; K = potassium; and OM = organic matter.

**Table 2:** Orthogonal design of fertilization treatments.

Treatment No.	Fertilization Treatments		
	RD (100%)		Biofertilizer (EM1) (mL)
	Inorganic Nitrogen (33.5%) (NH_4_NO_3_)	Organic Nitrogen (3%) (Chicken Manure)	
1	100%	0	0
2	100%	0	150 mL
3	75%	25%	
4	50%	50%	
5	25%	75%	
6	75%	25%	150 mL
7	50%	50%	150 mL
8	25%	75%	150 mL
9		100%	150 mL
10		100%	

RD = recommended dose.

**Table 3:** Effects of inorganic and organic nitrogen with EM1 extract on fruit weight (g), size (cm^3^/fruit), pulp, peel weight (g), and juice volume (cm^3^)/fruit of ‘Murcott’ mandarin in 2018 and 2019 seasons.

Fertilization Treatments			FW (g)		FS (cm^3^/fruit)		PW (g)		PEW (g)		Juice Volume (cm^3^)/fruit	
RD (100%)		EM1 (mL/tree)	1st Season 2018	2nd Season 2019	1st Season 2018	2nd Season 2019	1st Season 2018	2nd Season 2019	1st Season 2018	2nd Season 2019	1st Season 2018	2nd Season 2019
Inorganic N	Organic N											
100	0	0	161.8 d	199.0 de	157.0 e	188.0 d	135.2 cd	166.7 d	26.6 de	32.3 de	85.3 b	103.3 b
100	0	150	169.1 c	208.0 c	169.3 d	203.0 c	139.4 c	172.7 c	29.6 c	35.3 bc	81.0 c	101.0 b
75	25	0	184.1 a	226.4 a	191.7 a	228.7 a	151.8 a	187.2 a	32.3 a	39.2 a	94.7 a	109.5 a
50	50	0	159.7 de	196.4 e	169.7 d	228.7 a	134.2 d	165.5 de	25.5 e	30.9 e	80.3 c	96.7 c
25	75	0	158.5 de	195.0 e	157.0 e	187.7 d	131.2 de	161.8 ef	27.3 d	33.2 cde	74.3 d	89.3 d
75	25	150	166.5 c	204.8 cd	173.7 c	204.0 c	135.5 cd	167.1 d	31.0 abc	37.7 ab	74.3 d	89.3 d
50	50	150	176.4 b	217.0 b	187.0 b	215.8 b	145.1 b	179.0 b	31.4 ab	38.1 a	81.0 c	97.0 c
25	75	150	149.6 f	184.1 f	147.7 f	187.2 d	119.3 f	147.2 g	30.3 bc	36.9 ab	73.7 d	88.7 d
0	100	150	161.7 d	198.9 de	159.7 e	191.1 d	131.1 de	161.7 ef	30.6 bc	37.2 ab	69.5 e	83.7 e
0	100	0	156.8 e	192.9 e	158.3 e	189.7 d	129.1 e	159.2 f	27.7 d	33.7 cd	76.3 d	91.3 d

Mean values followed by the same letter(s) in each column are insignificantly different at *p* < 0.05.

**Table 4:** The impacts of different N fertilizers (organic and inorganic with EM1) on physiochemical properties of ‘Murcott’ mandarin in the 2018 and 2019 seasons.

Fertilization Treatments			T.S.S. (%)		TA * (%)		T.S.S/acid		Vit. C. (mg/100 mL)		NO_3_%	
RD (100%)		EM1 (mL/tree)	1st Season 2018	2nd Season 2019	1st Season 2018	2nd Season 2019	1st Season 2018	2nd Season 2019	1st Season 2018	2nd Season 2019	1st Season 2018	2nd Season 2019
Inorganic N	Organic N											
100	0	0	12.8 a	12.1 ab	1.3 a	1.2 ab	9.8 cdef	10.2 bc	40.2 de	40.8 c	0.46 a	0.48 a
100	0	150	11.0 b	11.2 bcd	1.1 cde	1.0 cd	10.0 bcd	11.2 b	50.6 a	41.4 c	0.43 a	0.44 b
75	25	0	10.7 bc	10.9 cd	1.2 abcd	1.3 a	8.7 def	8.8 c	44.0 bc	43.9 b	0.36 bc	0.39 c
50	50	0	9.5 cd	10.9 cd	1.1 de	1.2 abc	9.1 cdef	9.4 bc	45.1 b	41.7 c	0.41 ab	0.42 b
25	75	0	10.3 bcd	11.1 cd	1.3 ab	1.2 abc	8.0 f	9.6 bc	42.4 cd	39.5 d	0.33 cd	0.39 c
75	25	150	10.7 bc	10.9 cd	1.0 ef	1.0 d	10.7 bc	11.1 b	44.0 bc	45.3 a	0.47 a	0.50 a
50	50	150	9.7 d	10.8 d	1.2 bcde	1.1 bcd	8.1 ef	10.2 bc	41.8 cd	43.3 b	0.33 cd	0.38 c
25	75	150	12.5 a	11.8 abc	1.3 abc	1.3 a	9.9 bcde	9.2 c	43.5 bc	41.3 c	0.32 cd	0.35 d
0	100	150	13.2 a	12. 7 a	1.1 bcde	0.9 d	11.7 b	13.5 a	38.5 e	41.9 c	0.29 d	0.29 e
0	100	0	13.0 a	12.6 a	0.9 f	1.2 ab	14.7 a	10.4 bc	37.6 e	43.3 b	0.31 cd	0.34 d

* TA, total acidity. Mean values followed by the same letter(s) in each column are insignificantly different at *p* < 0.05.

**Table 5:** The impact of different N fertilizers (organic and inorganic with EM1) on total carbohydrate content of ‘Murcott’ mandarin fruit in 2018 and 2019 seasons.

Fertilization Treatments			Total Carbohydrates %		Total Sugars %		Reducing Sugars %		Non-Reducing Sugars %	
RD (100%)		EM1 (mL/tree)	1st Season 2018	2nd Season 2019	1st Season 2018	2nd Season 2019	1st Season 2018	2nd Season 2019	1st Season 2018	2nd Season 2019
Inorganic N	Organic N									
100	0	0	15.4 c	16.0 c	7.5 b	7.0 d	4.3 c	3.8 e	3.0 bcd	3.23 bcd
100	0	150	15.4 c	16.0 c	7.6 b	7.0 d	4.5 c	4.0 de	3.1 abc	2.94 d
75	25	0	15.6 c	16.2 c	7.9 b	7.3 d	4.6 c	4.1 de	3.6 ab	3.21 bcd
50	50	0	15.6 c	16.5 c	8.8 a	8.7 bc	6.0 ab	4.6 cd	2.8 cd	4.12 a
25	75	0	16.7 b	19.4 b	8.9 a	8.2 c	6.5 a	5.1 bc	2.4 d	3.06 cd
75	25	150	17.0 b	19.4 b	9.0 a	8.8 bc	5.4 b	5.1 bc	3.6 ab	3.71 abc
50	50	150	17.8 a	19.4 b	9.1 a	8.4 c	5.3 c	5.1 abc	3.8 a	3.27 bcd
25	75	150	17.9 a	19.4 b	9.3 a	8.2 c	6.0 ab	5.6 ab	3.4 abc	2.66 d
0	100	150	18.3 a	20.4 a	9.4 a	10.1 a	6.5 a	5.8 a	3.0 bcd	4.01 a
0	100	0	17.9 a	20.0 ab	9.4 a	9.4 b	6.5 a	5.6 ab	2.9 bcd	3.76 ab

Mean values followed by the same letter(s) in each column are insignificantly different at *p* < 0.05.

## Data Availability

The data presented in this study are available on request from the corresponding authors.
